# Myelin oligodendrocyte glycoprotein (MOG_35-55_)-induced experimental autoimmune encephalomyelitis is ameliorated in interleukin-32 alpha transgenic mice

**DOI:** 10.18632/oncotarget.6306

**Published:** 2015-11-11

**Authors:** Jaesuk Yun, Sun Mi Gu, Hyung Mun Yun, Dong Ju Son, Mi Hee Park, Moon Soon Lee, Jin Tae Hong

**Affiliations:** ^1^ Pharmacological Research Division, National Institute of Food and Drug Safety Evaluation (NIFDS), Ministry of Food and Drug Safety (MFDS), Osong-eup, Heungdeok-gu, Cheongju-si, Chungbuk, Republic of Korea; ^2^ College of Pharmacy and Medical Research Center, Chungbuk National University, Osong-eup, Heungdeok-gu, Cheongju-si, Chungbuk, Republic of Korea; ^3^ Department of Maxillofacial Tissue Regeneration, School of Dentistry and Research Center for Tooth and Periodontal Regeneration (MRC), Kyung Hee University, Dongdaemun-gu, Seoul, Republic of Korea; ^4^ College of Agriculture, Life and Environmental Sciences, Chungbuk National University, Heungdeok-gu, Cheongju-si, Chungbuk, Republic of Korea

**Keywords:** IL-32 alpha, experimental autoimmune encephalomyelitis, inflammation, multiple sclerosis, cytokines, Immunology and Microbiology Section, Immune response, Immunity

## Abstract

Multiple sclerosis (MS), also known as disseminated sclerosis or encephalomyelitis disseminate, is an inflammatory disease in which myelin in the spinal cord and brain are damaged. IL-32α is known as a critical molecule in the pathophysiology of immune-mediated chronic inflammatory disease such as rheumatoid arthritis, chronic pulmonary disease, and cancers. However, the role of IL-32α on spinal cord injuries and demyelination is poorly understood. Recently, we reported that the release of proinflammatory cytokines were reduced in IL-32α-overexpressing transgenic mice. In this study, we investigated whether IL-32α plays a role on MS using experimental autoimmune encephalomyelitis (EAE), an experimental mouse model of MS, in human IL-32α Tg mice. The Tg mice were immunized with MOG_35-55_ suspended in CFA emulsion followed by pertussis toxin, and then EAE paralysis of mice was scored. We observed that the paralytic severity and neuropathology of EAE in IL-32α Tg mice were significantly decreased compared with that of non-Tg mice. The immune cells infiltration, astrocytes/microglials activation, and pro-inflammatory cytokines (IL-1β and IL-6) levels in spinal cord were suppressed in IL-32α Tg mice. Furthermore, NG2 and O4 were decreased in IL-32α Tg mice, indicating that spinal cord damaging was suppressed. In addition, i*n vitro* assay also revealed that IL-32α has a preventive role against Con A stimulation which is evidenced by decrease in T cell proliferation and inflammatory cytokine levels in IL-32α overexpressed Jurkat cell. Taken together, our findings suggested that IL-32α may play a protective role in EAE by suppressing neuroinflammation in spinal cord.

## INTRODUCTION

MS is the most common inflammatory demyelinating disease of the central nervous system [1]. The disease typically presents between the ages of 20 and 40 and impacts approximately 35,000 individuals in the United States alone [1–3]. MS can lead to substantial disability with deficits seen in sensory, motor, autonomic, and neurocognitive function [2]. Its pathology is characterized by leukocyte infiltration, demyelination, oligodendrocyte loss, axonal transection, and a reactive astrogliosis [4, 5]. It is believed that early neurologic disability in MS is affected by conduction block in demyelinated axons, whereas axonal transection underlies the more permanent deficits observed later in the disease [6]. For study of MS, the most common animal model of experimental autoimmune encephalomyelitis (EAE) induction is currently based on the injection of an encephalitogenic peptide, MOG_35-55_ as well as proteolipid protein and myelin basic protein. The MOG_35-55_ peptide triggers chronic-progressive EAE in C57BL mice.

Interleukin-32 (IL-32) is a novel cytokine which was reported originally as a natural killer (NK) transcript 4 that was expressed in various human tissues and organs, such as spleen, thymus, leukocyte, lung, small intestine, colon, prostate, heart, placenta, liver, muscle, kidney, pancreas, and brain [7, 8]. The expression of IL-32 mRNA is more prominent in immune cells than in non-immune tissues [8–10]. IL-32 not only participates in host responses by induction of proinflammatory cytokines but also directly affects specific immunities differentiating monocytes into macrophage-like cell. It has been also reported that the increase of IL-32 expression correlates with clinical and histological markers of diseases such as rheumatoid arthritis (RA), suggesting the reduction of IL-32 activity may provide benefit to patients with RA [11]. In another study, IL-32 induced increase in constitutive IL-10 release, but a decrease in TNF-α and IL-6 [12]. IL-32 itself accounts for less inflammation in humans with ulcerative colitis (UC) [13]. Our previous study showed that IL-32 acts in decrease of pro-inflammatory cytokines and an increase of anti-inflammatory cytokines [14]. Thus, it is possible that IL-32 could act as both pro-inflammatory and anti-inflammatory cytokine under differential condition of disease.

In this work, we describe studies investigating the inflammatory role of IL-32 in an EAE mouse model, a widely accepted animal model of MS. Using IL-32 transgenic (Tg) mice, we demonstrate that IL-32 Tg mice displays reduced clinical and pathologic results.

## RESULTS

### Generation of IL-32α transgenic mice that display an decreased susceptibility to MOG_35-55_ -induced EAE

**Figure 1:**
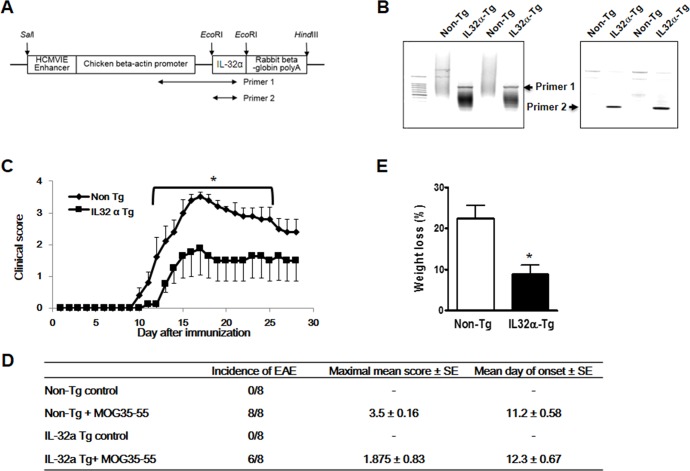
Production of IL-32α mice and MOG-induced EAE **A.** Transgene construct. cDNA of human IL-32α was inserted into a pCAGGs expression under the chicken beta-actin promoter. Arrows indicate PCR amplicon regions for IL-32α transgene confirmation **B.** Semi-quantitative PCR showed IL-32α expression in Tg mice. **C.** Immunization of non Tg and IL-32α Tg mice with MOG_35-55_ results in EAE. Data are shown as mean and standard error of the mean (*n* = 8). *p < 0.05 *vs* IL32α Tg (Student's *t*-test). **D.** Behavioral summary of EAE of non Tg and IL-32α mice. There are no significant differences. **E.** Weight loss induced by EAE was attenuated in IL-32α mice. *p < 0.05 *vs* non Tg (Student's *t*-test).

We generated human IL-32α-overexpressing transgenic mice (IL-32α mice) by subcloning IL-32α cDNA into the mammalian expression vector pCAGGS (Figure 1A). The success of procedure was confirmed by PCR of mouse tail genomic DNA using allele-specific primers (Figure 1B). The transgene was successfully transmitted to 50 % of pups from each littermate, as evaluated by genotyping. These founder mice were each back-crossed into the C57BL6/J background for eight generations. The male/female ratio was 50% for IL-32α transgenic and nontransgenic littermates.

Non-Tg and IL-32α mice sensitized at 8 weeks of age developed clinical signs of MOG_35-55_ peptide-induced EAE. The first paralysis appeared on day 10 in non-Tg group and day 11 in IL-32α group. Noteworthy differences were in the course of disease; non-Tg group's symptom is greater than that of IL-32α group (Figure 1C and 1D). Changes (between day 0 and day 28) of mean body weight (%) in non-Tg group were higher than IL-32α group (Figure 1E).

### EAE-induced spinal cord injury was reduced in IL-32α mice

**Figure 2:**
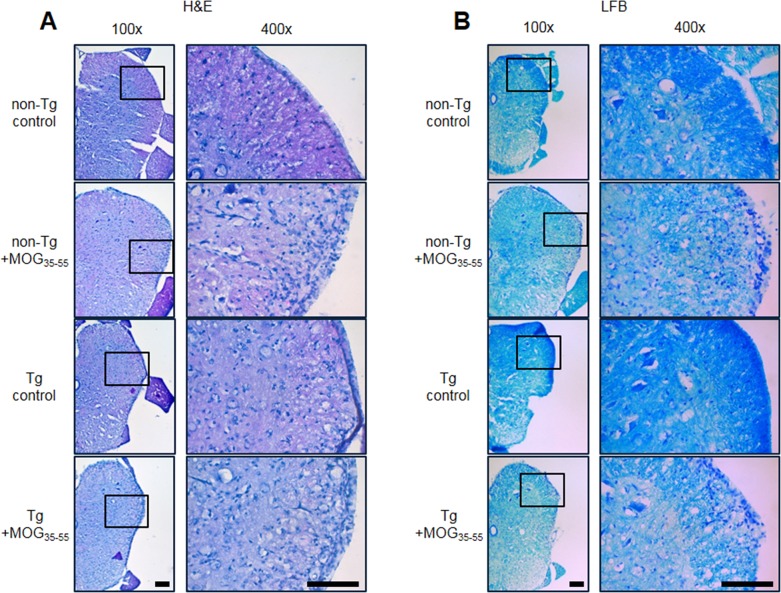
Representative spinal cord histopathology of MOG-induced EAE in non Tg and IL-32α mice **A.** MOG induced a prominent cellular infiltration of in the parenchyma adjacent to the pia mater in non Tg mice (second to top), but which was reduced in IL-32α mice (bottom). Sections were stained with hematoxylin-eosin. **B.** The cellular infiltration was associated with a loss of myelin. Sections were stained with Luxol fast blue. Scale bar: 100 μm.

To compare the lesion formation in the spinal cords of IL-32α mice group to that of non-Tg mice group, we used Hematoxylin and Eosin (H&E) staining for observing cell infiltration and Luxol Fast Blue (LFB) staining for observing demyelination. Using H&E staining in spinal cord sections, we found that mononuclear cell infiltration into the injured area during MOG_35-55_-induced EAE decreased in IL-32α mice group as compared to non-Tg mice group (Figure 2A). Furthermore, we observed that in the IL-32α mice group spinal cord sections significantly less reduction in LFB staining as compared to that of non-Tg mice group spinal cord sections, indicating that less demyelination occurred in the IL-32α mice spinal cord (Figure 2B).

### EAE-induced infiltration of immune cells was decreased in IL-32α mice

**Figure 3:**
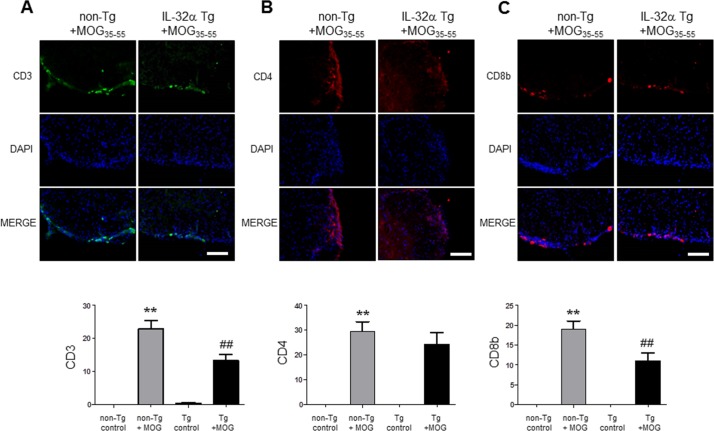
T cell infiltration in spinal cord of MOG-induced EAE in non Tg and IL-32α mice MOG induced a prominent **A.** CD3 and **C.** CD8b positive cell infiltration in the parenchyma adjacent to the pia mater in non Tg mice, but which was reduced in IL-32α mice. Data are shown as mean and standard error of the mean (*n* = 3). **B.** CD4^+^ cell infiltration was increased in non Tg mice, but there is no significant difference between non Tg and IL-32α mice. **p < 0.01 *vs*. non-Tg control, ##*p* < 0.01 *vs*. non-Tg + MOG (Twoway ANOVA followed by Bonferroni's test). Scale bar: 100 μm.

To analyse the profile of the infiltrating immune cells causing inflammation in the lesion area, we detected the expression of CD3^+^ (a maker of T cells), CD4^+^ (a maker of helper T cells), CD8b^+^ (a maker of cytotoxic T cells), CD11b^+^ (a maker of macrophages and microglials), F4/80^+^ (a maker of macrophages), CD16^+^ (a maker of NK cells) and CD19^+^ (a maker of B cells) by IF staining in the spinal cord sections. We found a massive elevation of immune cells (T cell, helper T cell, cytotoxic T cell, macrophage, NK cell and B cell) in the spinal cords of non-Tg mice (Figure 3 and Figure 8). However, IL-32α mice showed significant reduction in these immune cell numbers except CD4^+^ cells. These results show that MOG_35-55_ -induced immune response is reduced in IL-32α mice group.

### EAE-induced increase in inflammatory cytokine levels was inhibited in IL-32α mice

**Figure 4:**
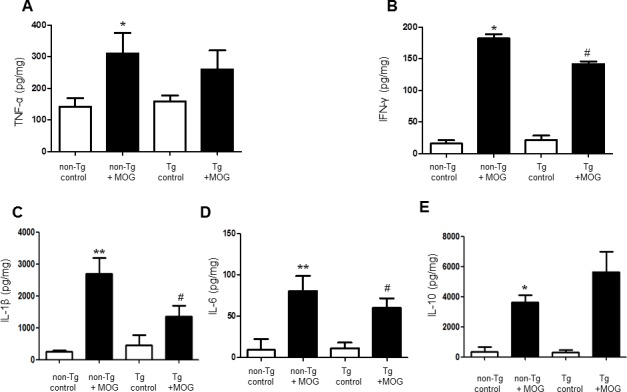
Cytokine profile of MOG-induced EAE in non Tg and IL-32α mice MOG induced a significant elevation of B. IFN-γ, **C.** IL-1β, and **D.** IL-6 levels in non Tg mice, but which was reduced in IL-32α mice. Data are shown as mean and standard error of the mean (*n* = 3). **A.** TNF-α and **E.** IL-10 was increased in non Tg mice, but there is no significant difference between non Tg and IL-32α mice. *p < 0.05, **p < 0.01 *vs*. non-Tg control, #*p* < 0.05 *vs*. non-Tg + MOG. (Twoway ANOVA followed by Bonferroni's test).

To measure cytokine levels related with EAE, we used ELISA kit by using spinal cord tissue lysis from control, non-Tg and IL-32α mice group. We observed that the level of inflammatory cytokines such as IL-1β, IFN-γ, and IL-6 was increased by MOG_35-55_ treatment in non-Tg, however, there is a significant difference between non-Tg + MOG and Tg + MOG groups (Figure 4B, 4C, and 4D). While, MOG-induced increase in TNF-alpha, and IL-10 levels were not affected in IL-32α mice group (Figure 4A and 4E). From this result, we suggest that the lower levels of cytokines in the spinal cords of the IL-32α EAE mice could simply be related to the reduced CNS immune cell infiltrates.

### EAE-induced increase in oligodendrocytes progenitor cell markers levels was attenuated in IL-32α mice

**Figure 5:**
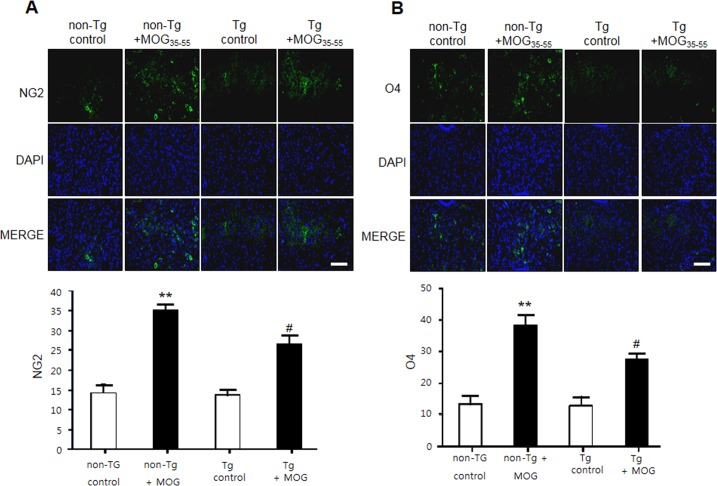
NG2 and O4 expression of MOG-induced EAE in non Tg and IL-32α mice MOG induced a significant elevation of **A.** NG2 and **B.** O4 expression levels in non Tg mice, but which was reduced in IL-32α mice. Data are shown as mean and standard error of the mean (*n* = 3). **p < 0.01 *vs*. non-Tg control, #*p* < 0.05 *vs*. non-Tg + MOG. (Twoway ANOVA followed by Bonferroni's test). Scale bar: 100 μm.

To observe the expression of oligodendrocytes and myelin, we also used the immunofluorescence staining to detect the expression of CNPase (myelinating oligodendrocytes marker), myelin basic protein (MBP), NG2 (a marker of oligodendrocyte progenitor cells;OPCs) and O4 (a marker of oligodendrocyte). CNPase and MBP were decreased in non-Tg mice by MOG treatment, however reduced CNPase and MBP levels were not rescued in IL-32α mice. In contrast, MOG-induced increase in NG2 and O4 levels were attenuated in IL-32α mice group (Figure 5, Figure 9A, and Figure 9B). Furthermore, we demonstrated that the expression levels of GFAP (a marker of astrocyte activation) and IBA-1 (a marker of microglia cell activation) were reduced in IL-32α mice group (Figure 9C and 9D).

### Con A-induced increase in cytokines levels was attenuated in IL-32α overexpressed Jurkat cells

**Figure 6:**
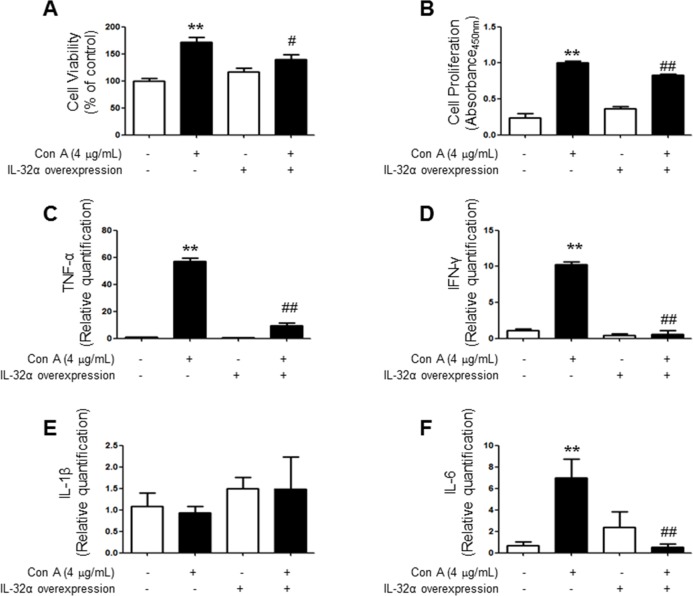
Effect of IL-32α overexpression on cytokine profile and pathogenicity of Con-A treated Jurkat cells Con-A induced a significant elevation of **A.** cell viability, **B.** proliferation, **C.** TNF-α, **D.** INF-γ, and **F.** IL-6 levels in Jurkat cells, but which was reduced by IL-32α overexpression. Data are shown as mean and standard error of the mean (*n* = 6). **E.** IL-1β was not affected with Con-A treatment. **p < 0.01 *vs*. Mock control, #*p* < 0.05, ##*p* < 0.01 *vs*. IL-32α + Con A. (Twoway ANOVA followed by Bonferroni's test). Scale bar: 100 μm.

To confirm the activation of T cells, we used the MTT assay for measuring cell viability and BrdU assay for measuring cell proliferation in Jurkat cells. We treated the con A (4 μg/mL) to stimulate the Jurkat cells. Cell viability was increased by treatment of con A and this effect was attenuated by IL-32α-overexpression (Figure 6A). Similarly, IL-32α-overexpression also reduced cell proliferation induced by con A (Figure 6B). To measure the level of inflammatory cytokine mRNAs, Jurkat cells were stimulated by same condition as cell viability and proliferation experiments, and then inflammatory cytokine levels were determined by real-time PCR. TNF-α, IFN-γ and IL-6 mRNA levels was increased in con A treated Jurkat cells, however those increase in cytokine levels was attenuated by IL-32α-overexpression. The significant changes of IL-1β mRNA levels were absent in all groups (Figure 6C - 6F).

### EAE-induced increase in cyclooxygenase 2 (COX-2) and inducible nitric oxide (iNOS) levels was attenuated in IL-32α mice

**Figure 7:**
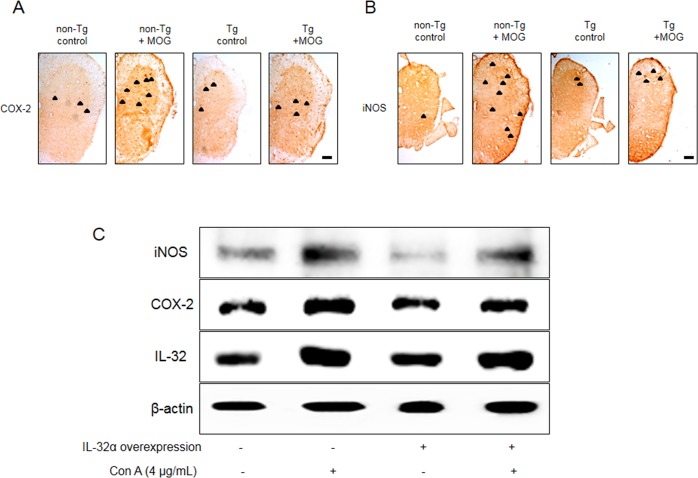
Representative COX-2 and iNOS expression patterns of IL-32α mice and IL-32α overexpressed Jurkat cells MOG increased a significant **A.** COX-2 and **B.** iNOS expression levels in non Tg mice (second to left), but which was reduced in IL-32α mice (right). Arrows indicate stained COX-2 and iNOS positive cells. Scale bar: 100 μm. **C.** Con A increased COX-2 and iNOS protein expression levels in control cells, but which was reduced in IL-32α overexpressed cells.

To measure the inflammatory response of EAE mice spinal cord, COX-2 and iNOS expression levels were visualized. We observed that the levels of COX-2 and iNOS were increased by MOG_35-55_ treatment in non-Tg, however, there is a significant difference between non-Tg + MOG and Tg + MOG groups (Figure 7A and 7B). We also demonstrated a similar result in Con A treated IL-32α overexpressed Jurkat cells (Figure 7C). These results show that MOG_35-55_ -induced inflammatory response is reduced in IL-32α group *in vitro* and *in vivo*.

**Figure 8:**
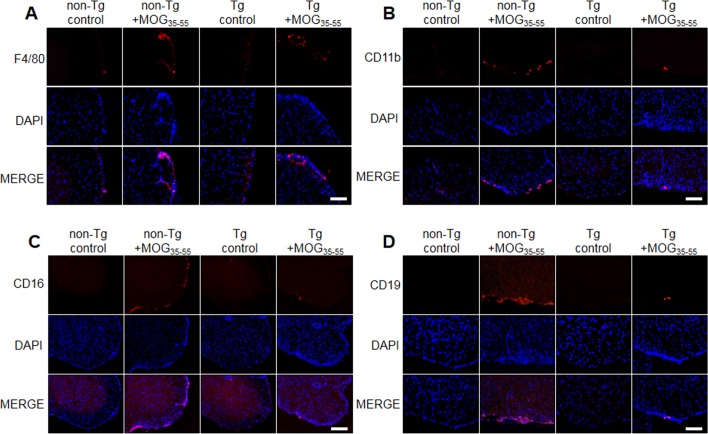
Representative spinal cord immune cell infiltration of MOG-induced EAE in non Tg and IL-32α mice MOG induced a prominent **A.** F4/80, **B.** CD11b, **C.** CD16, and **D.** CD19 positive cell infiltration in non Tg mice (second to left), but which was reduced in IL-32α mice (right). Scale bar: 100 μm.

**Figure 9:**
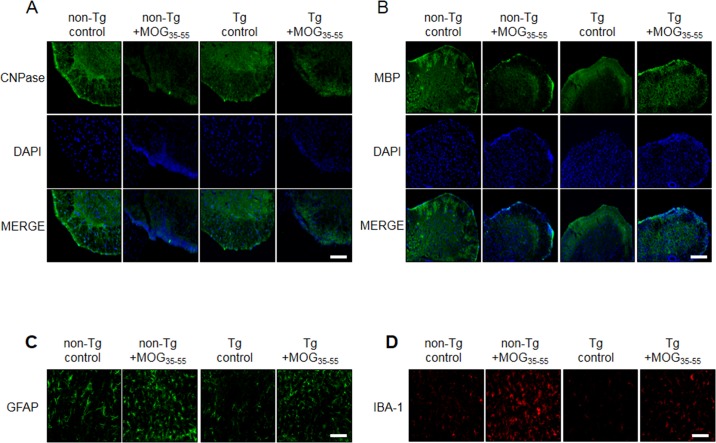
Representative spinal cord myelination related protein, astrocytes and microglia activation levels of MOG-induced EAE in non Tg and IL-32α mice MOG decreased **A.** CNPase and **B.** MBP expression levels in non Tg mice (second to left), but which was not attenuated in IL-32α mice (right). **C.** GFAP and **D.** IBA-1 expression levels were increased in non Tg mice (second to left), but which was attenuated in IL-32α mice (right). Scale bar: 100 μm.

## DISCUSSION

Various physiological and pathophysiological roles of IL-32 in immune response have been reported. In this study, EAE scores for paralysis is significantly decreased in parallel with reduced spinal injuries and infiltration of immune cells in IL-32α mice. We asked whether IL-32α mice showed reduction in proinflammatory cytokines production and demyelination of spinal cord. We demonstrated that cytokines were down regulated in IL-32α mice in comparison with those of non-Tg mice induced by EAE. Furthermore, NG2 and O4 were decreased in IL-32α mice, indicating that spinal cord damaging was suppressed. In addition, although the reduction of MBP and CNPase was not rescued in IL-32α mice, the demyelination was attenuated in IL-32α mice. These results suggest that reduction in inflammatory cytokines and demyelination may explain low EAE scores and spinal injuries in IL-32α mice. Meanwhile, IL-32α suppressed the infiltration of immune cells in spinal cord, as evidence by reduced number of CD3^+^, CD8^+^, B cell, NK cell and macrophages. Unexpectedly, CD4^+^ counts was not reduced in IL-32α mice, therefore, helper T cell may not be associated with the role of IL-32α role in EAE. This non-relationship between IL-32α and CD4^+^ may be further supported by previous study [15]. Furthermore, we demonstrated that the reduction of the astrocytes and microglials activation is related with the reduced inflammation in IL-32α mice. The expression levels of inflammatory marker COX-2 and iNOS in spinal lesion was also significantly reduced in IL-32α mice group. These anti-inflammatory-like actions of IL-32α are also observed in *in vitro* studies which employed IL-32α overexpressed Jurkat cells. Con A-induced increase in cell viability and proliferation was reduced by IL-32α overexpression. In addition, Con A-induced increase in TNF-α, IFN-γ, and IL-6 levels was decreased in IL-32α overexpressed Jurkat cells. Otherwise, the IL-1β production pattern was different in *in vivo* and *in vitro.* Even though the exact mechanism underlying those differences is not clear, MOG-induced T cell activation seems to be related with IL-1, but a case of Con-A is not [16]. These results suggest that the reduced infiltration of immune cells and inflammatory response may explain less severity of EAE in IL-32α mice.

In fact, IL-32 has been shown to exhibit properties typical of a proinflammatory cytokine and to drive the induction of other proinflammatory cytokines and chemokines, such as tumor necrosis factor-alpha (TNFα) and IL-1, IL-6, and IL-8. However, dual action of cytokines in autoimmune inflammatory demyelination is well known. For example, IFN-γ showed a paradoxical effect in IFN-γ-deficient mice [17]. Interestingly, we previously demonstrated that IL-32α transgenic mice showed the activation of signal transducer and activator of transcription 3 (STAT3) [18] which reduced inflammatory properties of type I IFNs [19]. Therefore, we can assume that IL-32α has protective effects via the downregulated IFN levels induced by STAT3 activation. In addition, IL-32 isoforms showed variable potency of cell death and cytokine production. Among IL-32 isoforms, IL-32γ has the most potent proinflammatory properties and it can be spliced into less active isoforms, IL-32β and IL-32α [20]. IL-32α is considered the least potent isoform in the process cell death and cell activation. Nonetheless, IL-32α has a benefit activity in cancer development. Our recent study [21] suggested that IL-32α suppressed colorectal cancer development in accordance with other study [22]. We did not elucidated a possible mechanism underlying the connection of IL-32α and other cytokines, IL-32α itself had no significant effects on immune cells infiltration and cytokines levels, rather reduced in inflammatory and severity of immune response models such as EAE and Con A treatment. These results suggest that IL-32α reduces inflammatory responses in elevated immune status of host. IL-32 also directly affects specific immunities differentiating monocytes into macrophage-like cells, therefore we could not exclude a possibility of direct action of IL-32α on immune cells activation. In rodent, the receptor for IL-32α is not clearly identified yet and IL-32α is considered an intracellular protein and may be released only after cell death. Therefore, we can assume that intracellular IL-32α in T cells interfere the immune activation/proliferation induced by elevated cytokines in EAE model. Intracellular signaling of IL-32α associated with immune cell activation is not clear, but tumor necrosis factor receptor 1 (TNFR-1) signaling was suggested as a target of IL-32α action by our previous work [21]. Because TNFR-1 stimulates dendritic cell maturation and CD8 T cell response [23], we suggest that IL-32α may reduce the immune cells activation via at least TNFR-1 signaling. Additional *in vivo* and *in vitro* models that are designed to study possible mechanism of IL-32α associated immune cell activation could be considered that might better reveal the function of IL-32α in EAE. In conclusion, our results suggested that IL-32α may suppress EAE by inhibition of neuroinflammation in spinal cord.

## MATERIALS AND METHODS

### Animals

IL-32α-transgenic mice were prepared as according to our previous report [18]. Animals were maintained under conventional housing conditions at 23 ± 2°C with a controlled 12 h light/dark cycle, and drinking water and rodent chow diet were provided *ad libitum* throughout the experiment. All experiments were approved and carried out according to the Guidelines for the Care and Use of Animals [Animal Care Committee of Chungbuk National University, Korea (CBNUA-436-12-02)]. All efforts were made to minimize animal suffering, to reduce the number of animals used.

### Induction and clinical evaluation of EAE

Non-Tg and IL-32α female mice (8 week old) were immunized with MOG_35-55_ peptide emulsified with complete Freund's adjuvant (CFA) using Hooke kits (Hooke laboratories, EK-0115, Lawrence, MA, USA) according to the manufacturer's instructions. In brief, 1 mg/mL of MOG_35-55_/CFA emulsion was injected subcutaneously into upper back and lower back of each 0.1 mL/animal (total 0.2 mL/animal). Two hours later, 2 μg/mL pertussis toxin (PTX) was intraperitoneally (i.p.) injected of each 0.1 mL/animal. Twenty four hours later, boosting shot of 2 μg/mL of PTX (0.1 ml/animal, i.p.) were given. Normal saline administrated non-Tg and IL-32α mice were used as vehicle control group. Mice were examined and scored daily for clinical signs of neurological deficit by a blinded investigator according to previous reports [24–26]. All other analyses were carried out on the 29th day.

### Jurkat cells culture

Jurkat cells (human prototypical CD28^+^ T cell leukemia, ATCC, Manassas, VA) were maintained with serum-supplemented culture media of Roswell Park Memorial Institute (RPMI) 1640 supplemented with FBS (10%) and penicillin (100 units/ml). The Jurkat cells were incubated in the culture medium in a humidified incubator at 37°C and 5% CO2. The cultured cells were treated simultaneously with concanavalin A (con A; 4 μg/mL) dissolved in distilled water.

### Construction of expression vectors and transfection

IL-32α cDNA was subcloned into pcDNA3.1 + 6×Myc vector using *E*coRI and *X*hoI. cDNAs for PKCδ and PKCε were subcloned into the pcDNA3.1 + 5×FLAG vector using *E*coRI and *X*hoI [27]. BCL6, SUMO-2, and ubiquitin cDNAs were PCR-amplified from a human spleen cDNA library (Clontech, Palo Alto, CA). The entire BCL6 gene was amplified and cloned into pcDNA3.1 + 5×FLAG vector using *E*coRI and *X*hoI. SUMO-2 and ubiquitin cDNAs were subcloned into the pCS3MT + 6×Myc vector using *E*coRI and *X*hoI. Jurkat cells were transfected with pcDNA3.1 + 5×FLAG-BCL6 and pCS3MT + -SUMO-2 or -ubiquitin using the Neon™ transfection system (Invitrogen, Carlsbad, CA) according to the manufacturer's instructions.

### Histological analysis

After transferred to 30 % sucrose solutions, spinal cords were cut into 18 μm sections by suing a cryostat microtome (Leica CM 1850; Leica Microsystems, Seoul, Korea). Spinal cord sections were stained for Luxol Fast Blue/Crystal Violet (LFB, IHC World, Ellicott City, MD) and Hematoxylin and Eosin (H&E) for identification of intact myelin and infiltrating cells respectively. Spinal cord sections were evaluated on a light microscopy (Olympus, Tokyo, Japan) (X50 or X200). For immunohistochemical analysis, the spinal cord sections were incubated for overnight at 4°C with a mouse polyclonal antibody against CNPase, MBP, NG2, O4 (1:200; Millipore, Billerica, MA, USA), CD3 and glial fibrillary acidic protein (GFAP) (1:200; Santa Cruz Biotechnology, Inc., Santa Cruz, CA, USA), a rat polyclonal antibody against F4/80 (1:100; Santa Cruz Biotechnology, Inc., Santa Cruz, CA, USA), CD4 (1:100, BD Biosciences, Franklin Lakes, NJ, USA), CD8b, CD11b and CD16/CD32 (1:100, eBioscince, San Diego, USA) a goat polyclonal antibody against ionize calcium-binding adapter molecule 1 (IBA-1) (1:300; Abcam, Inc., Cambridge, MA, USA), a rabbit polyclonal antibody against cyclooxygenase-2 (COX-2) (1:300; Cell Signaling Technology, Inc., Beverly, MA, USA) and inducible nitric oxide synthase (iNOS) (1:300; Novus Biologicals, Inc., Littleton). After incubation with the primary antibodies, spinal cord sections were incubated for 1-2 h at room temperature with an anti-rabbit, mouse, goat, or rat secondary antibody conjugated to Alexa Fluor 488 or 568 (Invitrogen-Molecular Probes, Carlsbad, CA, USA) or with the biotinylated goat anti-rabbit IgG-horseradish peroxidase (HRP) secondary antibodies (1:500; Santa Cruz Biotechnology, Inc., Santa Cruz, CA, USA). Images were acquired using an inverted fluorescent microscope (Axio Observer A1, Carl Zeiss, Oberkochen, Germany) (X 100 or 200) or a light microscopy (Microscope Axio Imager.A2, Carl Zeiss, Oberkochen, Germany) (×200).

### Western blotting

The Jurkat cells were sampled with lysis buffer (PRO-PREP^TM^, iNtRON Biotech, Daejeon, Korea) and gel electrophoresis was performed. The transfer membranes were incubated overnight at 4°C with iNOS (1:1000, Novus Biologicals, Inc., Littleton), COX-2 (1:1000, Cell Signaling Technology, Inc., Beverly, MA, USA), and β-actin (1:1000, Santa Cruz Biotechnology Inc. Santa Cruz, CA, USA) antibodies. The blots were then incubated with the corresponding conjugated goat anti-rabbit or goat anti-mouse IgG-horseradish peroxidase (HRP) (1:5000; Santa Cruz Biotechnology Inc. Santa Cruz, CA, USA) secondary antibodies. Immunoreactive proteins were detected with an enhanced chemiluminescence (ECL, Amersham Pharmacia Biotech) western blotting detection system.

### Measurement of cytokines

Lysates of spinal cord tissue were obtained through protein extraction buffer containing protease inhibitor. TNF-α, IFN-γ, IL-1β and IL-6 levels were determined according to the user's manual of ELISA Kit (R&D Systems, Minneapolis, MN, USA). The resulting color was assayed at 450 nm using a microplate absorbance reader (VersaMax ELISA, Molecular Devices, California, USA) after adding stop solution within 30 minutes.

### Cell viability assay

Mock vector expressed Jurkat cells and IL-32α overexpressed Jurkat cells were plated at a density of 1 × 10^4^ cells/well in 96-well plates per 200 μL medium and stimulated by con A (4 μg/mL) for 12 h. The cells were added to MTT [3-(4,5-dimethylthiazol-2-yl)-2,5-diphenyl-tetrazoliumbromide] solution (final concentration of 5 mg/mL) (Sigma, St. Louis, MO, USA) per 30 μL. After 2 h, MTT solution was removed, and the cells were added to dimethyl sulfoxide (DMSO) per 200 μL for 30 min. Finally, the resulting color was assayed at 540 nm using a microplate absorbance reader (VersaMax ELISA, Molecular Devices, California, USA).

### BrdU assay

Mock vector expressed Jurkat cells and IL-32α overexpressed Jurkat cells were plated at a density of 1 × 10^4^ cells/well in 96-well plates per 200 μL medium and stimulated by con A (4 μg/mL) for 12 h. Detection of BrdU incorporation was performed by ELISA (BrdU Cell Proliferation Assay Kit, Cell Signaling Technology, Danvers, MA, USA) according to the manufacturer's instructions.

### Quantitative real-time PCR

Jurkat cells and IL-32α overexpression Jurkat cells were plated at a density of 3 × 10^6^ cells/well in 60 Φ plates per 200 μL medium and stimulated by con A (4 μg/mL) for 2 h. For mRNA quantification, total RNA was extracted using the easy-BLUR^TM^ total RNA extraction kit (iNtRON Biotech, Daejeon, Korea). The cDNA was synthesized using High Capacity cDNA Reverse Transcription Kits (Applied Biosystems, Foster city, CA) according to the manufacturer's protocol. Quantitative real-time PCR was performed on cDNA using Brilliatn III Ultra-Fast Green QPCR Master Mix (Agilent Technologies, Waldbronn, Germany) with primers (Bioneer, Daejeon, Korea), specific for β-actin (N1080), TNF-α (N-1072), IFN-γ (N-1055), IL-1β (N-1058) and IL-6 (N-1063). All reverse transcription reactions were run in a StepOnePlus Real-Time PCR System (Applied Biosystems, Foster city, CA). For the calculation of relative quantification, the 2^−ΔΔCT^ formula was used, where: -ΔΔCT = (C_T,target_ − C_T,beta-actin_) experimental sample − (C_T,target_ − C_T,beta-actin_) control sample.

### Statistical analysis

Statistical analysis of the data was carried out using Student's t-test or two-way ANOVA followed by Bonferroni's post-hoc analysis using GraphPad Prism 5 software (Version 5.01, GraphPad software, Inc., La Jolla, USA). The numbers and percentage of immune cells in each picture were then counted using the ImageJ.
